# Long-term outcomes of paediatric craniopharyngiomas: a comparison of two large international series

**DOI:** 10.1007/s00381-026-07154-7

**Published:** 2026-02-20

**Authors:** Vitor Nagai Yamaki, Jai Sidpra, Bruno Santanna Peres, Valentina Lind, Catuto Domingos Alexandre Quianga, Guilherme Jose da Costa Borsatto, Joao Paulo Mota Telles, Inês Nobrega Silva, Juan Pedro Martinez-Barbera, Sniya Sudhakar, Asthik Biswas, Kshitij Mankad, Hoong-Wei Gan, Hani J. Marcus, Dominic Thompson, Noor ul Owase Jeelani, Darren R. Hargrave, Hamilton Matushita, Suely Kazue Nagahashi Marie, Kristian Aquilina

**Affiliations:** 1 Department of Paediatric, Neurosurgery/Institute of Child Health, Great Ormond Street Hospital for Children, University College London (UCL), London, UK; 2 Department of Neurosurgery, Hospital das Clínicas da Faculdade de Medicina da Universidade de Sao Paulo, Sao Paulo, Brazil; 3 Developmental Biology and Cancer Section, University, College London Great Ormond Street Institute of Child Health, London, UK; 4 Department of Radiology, Hospital, das Clinicas da Faculdade de Medicina da Universidade de Sao Paulo, Sao Paulo, Brazil; 5 Department of Neurosurgery, National Hospital for Neurology and Neurosurgery, London, UK; 6 Department of Neurology, Laboratory of Molecular and Cellular, LIM 15, Faculdade de Medicina FMUSP, Universidade de Sao Paulo, Brazil; 7 Department of Neuroradiology, Great Ormond Street Hospital for Children, NHS Foundation Trust, London, UK; 8 Department of Endocrinology, Great Ormond Street Hospital for Children, NHS Foundation Trust, London, UK; 9 Department of Oncology, Great Ormond Street Hospital for Children, NHS Foundation Trust, London, UK

**Keywords:** Craniopharyngioma, Hypothalamic diseases, Diabetes insipidus, Cognitive outcomes

## Abstract

**Purpose:**

Adamantinomatous craniopharyngioma (ACP) is an uncommon and anatomically variable tumor in children. The balance between critical treatment objectives, including the pursuit of gross total surgical resection and the reduction of hypothalamic injury and tumor recurrence, remains difficult and controversial. In this retrospective observational study, we compare the management and outcome of ACP in two large paediatric neurosurgical centres to determine how variations in tumor characteristics and management influence long-term outcomes.

**Methods:**

In this retrospective observational study, consecutive children (aged ≤ 18 years) diagnosed with primary ACP between 1997 and 2023 at two tertiary paediatric neurosurgical centres (Great Ormond Street Hospital (GOSH), United Kingdom, and Universidade de São Paulo (USP), Brazil) were evaluated. Functional outcomes related to pituitary function, hypothalamic injury, cognition, and vision were analysed. Scans were independently reviewed for tumor size, characteristics, and relationship to the optic apparatus and hypothalamus. Extent of surgery was documented.

**Results:**

123 patients (USP = 52; GOSH = 71) were included. Mean follow-up was 10.1 ± 5.6 years at USP and 7.5 ± 4.8 years at GOSH. There were no demographic differences. Rates of growth hormone deficiency (USP = 62.5%; GOSH = 35%; *p* = 0.009) and visual deficits (*p* < 0.05) were higher in the USP cohort at initial presentation. Patients at GOSH presented with predominantly cystic lesions (*n* = 46/60; 76.7%) while at USP solid tumors (*n* = 24/32; 75%) (*p* = 0.05) were more prevalent. Paris grade for hypothalamic involvement was higher in the GOSH cohort (*p* = 0.01). Although gross total resection (GTR) was similar in the two groups, cyst aspiration plus radiotherapy was commoner at GOSH, whereas debulking was commoner at USP (*p* < 0.001). Post-treatment hypothalamic scores were better in the GOSH cohort (*p* < 0.04). Patients at GOSH had earlier recurrences (25.5 ± 32 months) compared to patients treated at USP (37.4 ± 59.5 months) (*p* = 0.01). In multivariate analysis, STR/GTR (OR 0.17; 95%CI:0.05–0.55, *p* < 0.01) and older age at diagnosis (OR 0.91, 95%CI:0.82–0.99, *p* = 0.04) were associated with longer progression-free survival.

**Conclusion:**

Comparative analysis of two large series of ACP in children identified different paradigms of surgical management driven by distinct clinical–radiological presentations. While cyst aspiration plus radiotherapy protects against hypothalamic injury, this may occur at the cost of earlier tumour recurrence. Further studies to define this balance are urgently needed.

**Previous presentations:**

21st International Symposium on Pediatric Neuro-Oncology.

June 29–July 2, 2024

## Introduction

Adamantinomatous craniopharyngioma (ACP) accounts for approximately 80% of childhood hypothalamic and pituitary [1–3] tumours [4]. As expected for a low-grade tumour, overall survival is excellent, and has been reported as high as 92% at 10 years [5]. However, tumour proximity to critical structures, including the visual pathways, arteries, hypothalamus, and pituitary gland can result in significant, often iatrogenic, long-term neurologic and endocrine morbidity, in particular hypothalamic, pituitary, and cognitive dysfunction [5–7].

Informed neurosurgical decision-making reduces morbidity and improves patient outcomes[1, 8]. However, there is considerable variability in neurosurgical management paradigms for ACP. Gross total resection (GTR) decreases the chance of recurrence but is the most significant risk factor for developing hypothalamic syndrome[1, 9]. Conversely, less invasive approaches may allow better preservation of hypothalamic function but often require adjuvant radiotherapy (RT), which increases the risk of late hypopituitarism, vascular abnormalities, and cognitive impairment[3, 5, 6, 10].

Given the complexity of ACP management, achieving optimal outcomes requires care from an experienced multidisciplinary team. Evidence suggests that centralisation of care in specialised centres can improve treatment strategies and long-term outcomes[11]. However, current evidence for ACP management and outcomes is primarily derived from single-institution case series with limited sample sizes and follow-up. In the context of rare and highly variable pathologies, such as paediatric ACP, where there are difficult challenges to develop randomised clinical trials, a direct analysis and comparison of management and outcomes in two cohorts from two institutions is useful.

This retrospective observational study aims to compare the long-term outcomes of ACPs treated at two large paediatric neurosurgery centres. By delineating differences in tumour presentations and management paradigms, the study seeks to identify factors associated with prognosis and morbidity, which can inform future clinical guidelines and trial design.

## Material and methods

This retrospective observational study included consecutive children (aged ≤ 18 years) diagnosed with primary ACP between 1997 and 2023 at two tertiary paediatric neuro-oncology referral centres (Great Ormond Street Hospital (GOSH), United Kingdom, and Universidade de São Paulo (USP), Brazil), in accordance with the STROBE[12] statement for observational research. Patients were identified from prospectively maintained operative databases at each study site. Patients diagnosed with papillary craniopharyngioma were excluded. Minimal follow-up for inclusion was 6 months.

**Fig. 1:**
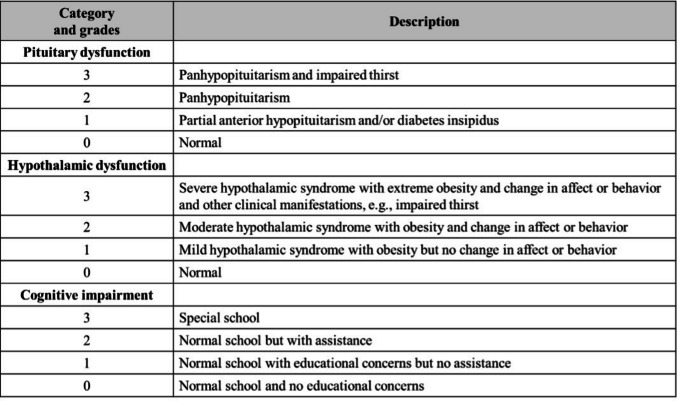
Grading system used to assess morbidity*. *Thompson et al. (2005)[13]

A comparative analysis of the long-term outcomes of childhood ACP managed in two high-volume paediatric neurosurgical centres was carried out. Functional outcomes data were collected and analysed across four distinct domains: (1) pituitary function; (2) hypothalamic syndrome; (3) cognitive function; and (4) visual outcomes (acuity and visual field deficits) according to a previously reported evidence-based grading system from our institution (Fig. 1) [13]. Data collection was standardised and recorded across both institutions to avoid bias.

Demographic, clinical presentation, treatment modality, and outcome data were collected through retrospective review of the electronic health record during first admission, at the time of surgery, immediate post-operative period, at 5-year follow-up, and/or at the last clinical appointment. To additionally assess hypothalamic syndrome, obesity was defined using body mass index (BMI) cut-off points per age, as defined by Cole et al.[14]. Brain MRI scans from both units were reviewed by two board-certified paediatric neuroradiologists to classify tumours according to the main tumour component (solid or cystic) and to the location of the solid component. In relation to the optic chiasm, tumours were classified as retrochiasmatic, prechiasmatic, or both; according to the relation of tumours to the sellar region, tumours were classified as suprasellar, sellar, or infrasellar. With respect to hypothalamic invasion, the Paris classification was used[15] (grade 0: no hypothalamic invasion; grade 1: hypothalamus displaced by the tumour; grade 2: hypothalamic involvement). Baseline MRI scans were segmented on volumetric T1-weighted images by a board-certified paediatric neuroradiologist using ITK-SNAP, defining both solid and cystic components, as well as the whole tumour.

Results of surgical interventions were categorised by the same board-certified paediatric neuroradiologists according to the extent of resection of solid and cystic components, as: cyst aspiration (CAsp), subtotal resection (STR), and gross total resection (GTR). Any surgical approach to the tumour was classified as STR if the post-operative MRI showed a solid tumour resection greater than 10%; GTR was defined as no solid tumour visible on post-operative MRI. If more than one type of surgery was performed within a 3-month period, all were recorded, and the most invasive resection was used to assign a categoric class.

Recurrence was defined according to the Response Assessment in Paediatric Neuro-oncology (RAPNO) Craniopharyngioma working group criteria for solid and cystic lesions [16]. Recurrence was also analysed in a subgroup of patients who received radiotherapy (RT) either as adjuvant treatment—within 90 days of the initial surgical intervention—or as a subsequent treatment, initiated more than 90 days after surgery.

### Statistical analysis

Normal (Gaussian) distribution was confirmed using the Shapiro-Wilk test. Normally distributed continuous data are presented as the mean ± standard deviation whilst other distributions are reported as the median (interquartile range). Categoric data are presented as a percentage frequency. Chi-squared, *t*-tests, or Mann-Whitney *U*-tests were employed for comparison of groups, as appropriate. Linear regression models were fitted to evaluate possible predictors of continuous outcomes, while logistic regression models were modelled to evaluate possible predictors of binary outcomes. Kaplan-Meier curves were fitted for time-to-event analyses. *p* < 0.05 was considered significant throughout and hypotheses were two-tailed. Analyses were performed using R (R Foundation for Statistical Computing, Vienna, Austria, 2021) and SPSS (SPSS for Windows, Version 16.0. Chicago, SPSS Inc.).

## Results

### Demographics and clinical presentation

This study includes 123 patients in the period 1997–2023 across both centres (USP = 52; GOSH = 71). No significant demographic differences were identified between study sites (Table 1). The mean follow-up was 10.1 ± 5.6 years at USP and 7.5 ± 4.8 years at GOSH. Headache (USP: *n* = 44/71; 75%/GOSH: *n* = 30/40; 62%) and visual deficits (USP: *n* = 24/40; 60%/GOSH: *n* = 29/71; 40.8%) were the most common presenting symptoms. There were statistically significantly higher rates of growth hormone deficiency (USP = 62.5%; GOSH = 35%; *p* = 0.009) and visual deficits in the USP cohort at initial presentation (Table 1).

**Table 1:** Demographics and initial clinical presentation of patients with craniopharyngiomas from the two neurosurgery units

Demographics	GOSH (*N* = 71)	USP (*N* = 52)	*p*-value
Gender (female) Age (years)	46.5% (*n* = 33/71) 8.2 ± 4.1	57.7% (*n* = 30/52) 9.2 ± 4.6	- -
Clinical presentation (GOSH: *n* = 71/USP: *n* = 41/52)*			
Headache	62% (*n* = 44/71)	75% (*n* = 30/40)	*p* = 0.2
Visual field deficits	38% (*n* = 27/71)	60% (*n* = 24/40)	*p* = 0.02
Visual acuity deficits	40.8% (*n* = 29/71)	60% (*n* = 24/40)	*p* = 0.04
Growth deficiency	35.2% (*n* = 25/71)	62.5% (*n* = 25/40)	*p* = 0.05
Diabetes insipidus	12.7% (*n* = 9/71)	22.5% (*n* = 9/40)	*p* = 0.14
Strabismus (cranial nerve paralysis)	9.9% (*n* = 7/71)	10% (*n* = 4/40)	*p* = 0.6
Acute hydrocephalus	11.3% (*n* = 8/71)	7.5% (*n* = 3/40)	*p* = 0.3

*GOSH*, Great Ormond Street Hospital/*USP*, Universidade de São Paulo

^*^Denominators vary due to missing data; *n*/*N* shown per variable

### Radiological characteristics

Ninety-two children had pre-operative brain MRI available for central neuroimaging review (GOSH: *n* = 60/USP: *n* = 32). Radiological characteristics are summarized in Table 2. Patients at GOSH presented with predominantly cystic lesions (*n* = 46/60; 76.7%), while at USP, there were predominantly solid lesions (*n* = 24/32; 75%), *p* = 0.05. Whilst there were no differences in the tumour’s diameters, volumetric analysis revealed a non-significant trend for a larger volume of the cystic component in the USP cohort (solid component: USP = 6723.5 ± 7879.6 mm^3^/GOSH = 5412.2 ± 6837.8 mm^3^; *p* = 0.20/cystic component: USP = 37,354.5 ± 63,555 mm^3^/GOSH = 22,098.7 ± 32,245.8 mm^3^; *p* = 0.02).

**Table 2:** Radiological characteristics of craniopharyngiomas from the two neurosurgery units

	GOSH (***n*** = 60/71)*	USP (***n*** = 32/51)*	***p***
Tumour characteristics			
Predominantly cystic	76.7% (*n* = 46/60)	25% (*n* = 8/32)	*p* = 0.05
Predominantly solid	23.3% (*n* = 14/60)	75% (*n* = 24/32)	
Mean size (mm)			
Cystic	36.5 ± 19.3 mm	40.0 ± 22.0 mm	*p* = 0.1
Solid	18.8 ± 10.8 mm	18.2 ± 10.1 mm	*p = 0.5*
Whole tumour	43.3 ± 16.6 mm	45.0 ± 20.3 mm	*p = 0.1*
Mean volume (mm^3^)			
Cystic	22,098.7 ± 32,245.8 mm^3^	37,354.5 ± 63,555 mm^3^	*p* = 0.02
Solid	5412.2 ± 6837.8 mm^3^	6723.5 ± 7879.6 mm^3^	*p = 0.20*
Whole tumour	27,511.0 ± 32,950.0 mm^3^	44,078.0 ± 63,542.6 mm^3^	*p = 0.01*
Location of solid component			
Suprasellar	1.2% (*n* = 1/60)	3.1% (*n* = 1/32)	*p* = 0.5
Sellar	38.3% (*n* = 23/60)	43.7 (*n* = 14/32)	
Infrasellar	46.7% (*n* = 28/60)	40.6% (*n* = 13/32)	
Retrochiasmatic	66.6% (*n* = 40/60)	59.3% (*n* = 19/32)	*p* = 0.3
Prechiasmatic	21.6% (*n* = 13/60)	34.3% (*n* = 11/32)	
Both	10% (*n* = 6/60)	6.2% (*n* = 2/32)	
Paris classification			
Grade 0	15% (*n* = 9/60)	31.2% (*n* = 10/32)	*p* = 0.01
Grade 1	18.3% (*n* = 11/60)	31.2% (*n* = 10/32)	
Grade 2	66.6% (*n* = 40/60)	37.5% (*n* = 12/32)	
Peritumoral T2-hyperintesity	48.3% (*n* = 29/60)	31.2% (*n* = 10/32)	*p* = 0.08
Ventriculomegaly	45% (*n* = 27/60)	43.7% (*n* = 14/32)	*p* = 0.5

*GOSH*, Great Ormond Street Hospital/*USP*, Universidade de São Paulo

^*^Denominators vary due to missing data; *n*/*N* shown per variable

The location of the solid component was predominantly in the sellar/suprasellar regions (USP: 84.3%; *n* = 27/32; GOSH: 85%; *n* = 55/60) and predominantly retrochiasmatic (USP: 59.3%; *n* = 19/32; GOSH: 66.6%; *n* = 40/60). With regard to hypothalamic involvement, using the Paris classification[15], there was equal distribution within the three grades of hypothalamic involvement in the USP cohort (grade 0: *n* = 10/60; 31.2%; grade 1: *n* = 10/60; 31.2%; grade 2: *n* = 12/60; 37.5%). In the GOSH series, there was a significantly (*p* = 0.01) higher rate of hypothalamic invasion (grade 2: *n* = 40/60; 66.6%) or displacement (grade 1: *n* = 11/60; 18.3%). 

### Management of childhood craniopharyngiomas

In the USP cohort, surgical debulking was the predominant initial treatment (*n* = 49/52; 94.2%) compared with *n* = 27/71 (38%) in the GOSH cohort. There were higher rates of subtotal resection in the USP patients (USP: *n* = 41/52; 78.8%; GOSH: *n* = 20/71; 28.1%; *p* < 0.001); in the GOSH cohort, there were higher rates of cyst aspiration alone (USP: *n* = 3/52; 5.8%/GOSH: 44/71; 62%; *p* < 0.001).

**Fig. 2:**
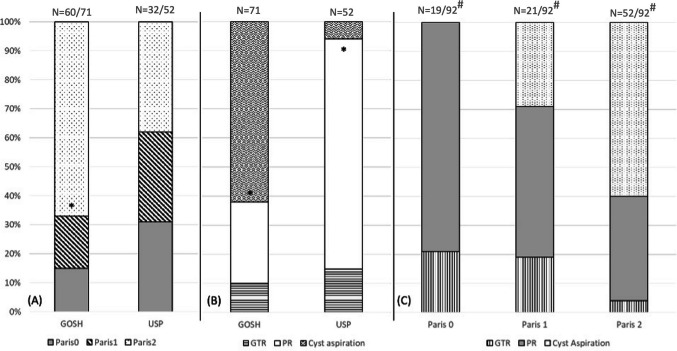
Management of CP at GOSH and at USP according to the Paris classification[15] of hypothalamic involvement of the tumours. **A** Hypothalamic involvement in the two cohorts according to Paris classification. **B** Surgical strategy for patients with ACP in the two cohorts. **C** Overall surgical approach according to Puget classification of hypothalamic involvement. *p < 0.05. N: numbers above bars indicate total N per group. #: Denominators vary due to missing data

GTR rates were similar between two series (USP: *n* = 8/52; 15.4%; GOSH: *n* = 7/71; 9.9%) (Fig. 2). Transphenoidal approach was only performed in 10 (*n* = 10/71; 14%) patients at GOSH; GTR was achieved in 30% of those patients (*n* = 3/10). All procedures at the USP cohort were performed transcranially.

Proton beam therapy (PBT) was only available for patients at GOSH from 2011. Other patients underwent conventional radiotherapy in both centres. 80% (*n* = 57/71) of patients at GOSH received RT (PBT: *n* = 39/57–68.4%; conventional fractioned radiotherapy: *n* = 18/57–31.6%); compared to 32% (*n* = 17/52) at USP (*p* < 0.05). Time to radiotherapy also differed between the two series as a less invasive surgical approach necessitated RT as part of the initial treatment. The average timing for RT at GOSH was 8.5 ± 13.6 months, whilst at USP was 23.8 ± 28.5 months (*p* < 0.001).

### Long-term clinical outcomes

Differences in clinical and radiological presentation led to distinct management strategies at USP and GOSH. Surgical approaches at GOSH were generally more conservative, with a higher proportion of cyst aspirations followed by radiotherapy, whereas at USP surgery more often aimed at achieving gross-total resection to avoid radiotherapy. There were no significant differences in cohort demographics between the two centres. Mean follow-up at GOSH was 7.5 ± 4.8 years compared to 10.1 ± 5.6 years at USP (*p* < 0.05). Of note, patients at USP were followed up until early adulthood, while at GOSH most patients were transitioned to adult neurosurgical services at 16 to 18 years of age. Therefore, interpretation of comparative outcomes should take into account this difference as outcomes were measured at the last clinical appointment available.

No differences in endocrine outcomes were identified using our classification between patients at GOSH and at USP (p > 0.05). Analysing individual hormones replaced, there were no differences in cortisol, gonadotrophins, or desmopressin (DDAVP) replacement; however, 80% (*n* = 52/65) of patients received growth hormone replacement at GOSH, compared to 57.1% (*n* = 24/42) of patients at USP (*p* < 0.05). A similar finding was identified for levothyroxine replacement (GOSH: 86.1%, *n* = 56/65; USP: 66.7%, *n* = 28/42; *p* < 0.05). It is important to mention that there is a nearly 2-year difference between mean follow-ups at GOSH and USP.

**Fig. 3:**
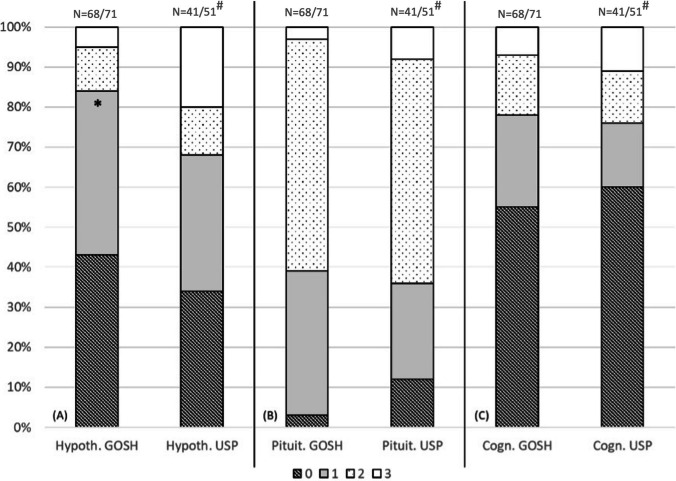
Morbidity of craniopharyngioma management at GOSH and USP based on GOSH grading system[13]. **A** Comparative analysis of long-term hypothalamic scores between two centres. **B** Comparative analysis of long-term pituitary scores between two units. **C** Comparative analysis of long-term cognitive scores between two centres. Hypoth., hypothalamic/Pituit., pituitary/Cogn., cognitive/*p < 0.05. N: numbers above bars indicate total N per group. #: Denominators vary due to missing data

With regards to the hypothalamic outcomes, patients treated at GOSH had more favourable hypothalamic scores compared to the USP cohort. 83.8% (*n* = 57/68) of patients at GOSH had normal or mild hypothalamic syndrome features (scores 0 or 1) while the comparable figure for USP was 68.2% (*n* = 28/41). For patients with more severe hypothalamic injury (severe obesity, behavioural changes, and other clinical manifestations), there were three (4.4%; *n* = 3/68) patients at GOSH and 8 (19.6%; *n* = 8/41) patients at USP (*p* < 0.039). There were no differences in cognitive outcomes between the two cohorts (*p* = 0.8) (Fig. 3).

**Fig. 4:**
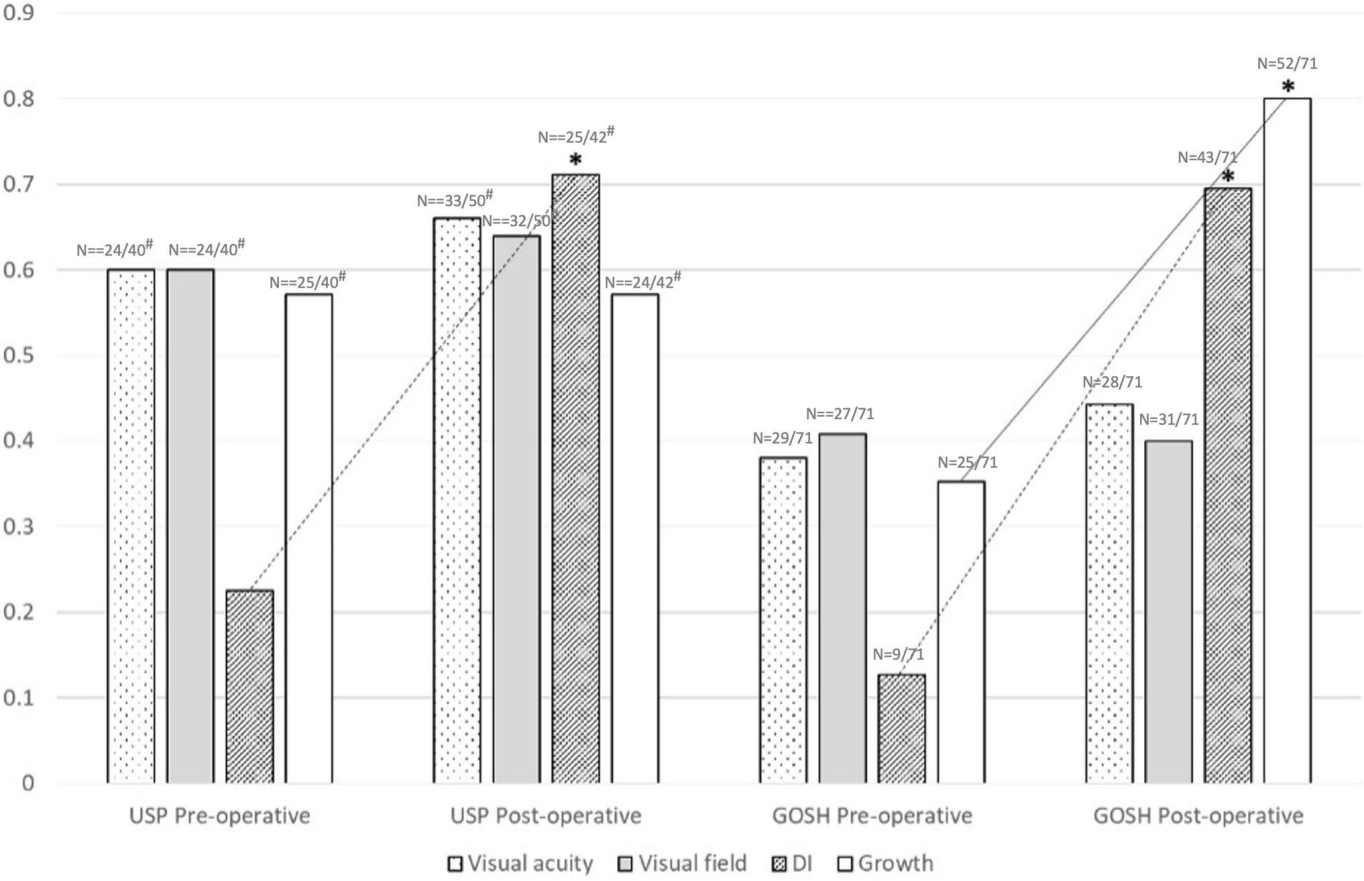
Comparative analysis of pre- and post-operative long-term outcomes in the two neurosurgery centres in relation to deficits in visual acuity and field, diabetes insipidus, and growth hormone requirement. (USP ≈ 10–years/GOSH ≈ 7.5–years follow-up). *p < 0.05/DI, diabetes insipidus/growth − requirement for growth hormone replacement. N: numbers above bars indicate total N per group. #: Denominators vary due to missing data

At last follow up, there was no significant improvement in baseline visual function in either cohort. Patients at USP presented with worse visual function at baseline and, therefore, worse outcomes in the long term (*p* < 0.05). In the USP cohort, the incidence of DI rose from 22.5% (*n* = 9/40) pre-operatively to 59.5% (*n* = 25/42) in the post-operative period, an absolute increase of 37.0 percentage points; while in the GOSH cohort, DI frequency increased from 12.7% (*n* = 9/71) to 66.2% (*n* = 43/65), an increase of 53.3 percentage points. Regarding growth hormone deficiency, it remained nearly unchanged at USP (pre-operative: 62.5%; post-operative: 57.1%; −5.4 percentual points); but with an absolute increase of 44.8 percentage points at the GOSH cohort (35.2% pre-operative to 80% post-operative) (Fig. 4).

Long-term outcomes are summarised in Table 3.

**Table 3:** Long term outcomes of patients with craniopharyngiomas from the two neurosurgery units

	GOSH	USP	***p***
Visual acuity deficit	40% (*n* = 28/70)	64% (*n* = 32/50)	*p* = 0.008
Visual field deficit	44.3% (*n* = 31/70)	66% (*n* = 33/50)	*p = 0.015*
Insipidus diabetes(GOSH: *n* = 70/71;USP: *n* = 50/52)*	68.5% (*n* = 48/70)	74.0% (*n* = 37/50)	*p = 0.5*
Pituitary outcome (GOSH: *n* = 69/71; USP: *n* = 41/52)*			
0	2.9% (*n* = 2/69)	12.2% (*n* = 5/41)	*p* = 0.125
1	36.2% (*n* = 25/69)	24.4% (*n* = 10/41)	
2	58% (*n* = 40/69)	56.1% (*n* = 23/41)	
3	2.9% (*n* = 2/69)	7.3% (*n* = 3/41)	
Long-term hormone deficiency (GOSH: *n* = 65/71; USP: *n* = 42/52)*			
Cortisol	75.4% (*n* = 49/65)	62% (*n* = 26/42)	*p* = 0.10
Growth hormone	80% (*n* = 52/65)	57.1% (*n* = 24/42)	*p = 0.010*
Thyroid	86.1% (*n* = 56/65)	66.7% (*n* = 28/42)	*p = 0.016*
Gonadotrophins	64.6% (*n* = 42/65)	65.8% (*n* = 27/42)	*p = 0.5*
Insipidus diabetes	69.5% (*n* = 48/69)	71.1% (*n* = 37/52)	*p = 0.5*
Hypothalamic outcome (GOSH: *n* = 68/71; USP: *n* = 41/52)*			
0	42.6% (*n* = 29/68)	34.1% (*n* = 14/41)	*p* = 0.039
1	41.2% (*n* = 28/68)	34.1% (*n* = 14/41)	
2	11.8% (*n* = 8/68)	12.2% (*n* = 5/41)	
3	4.4% (*n* = 3/68)	19.6% (*n* = 8/41)	
Cognitive outcomes (GOSH: *n* = 65/71; USP: *n* = 44/52)*			
0	55.4% (*n* = 36/65)	59% (*n* = 26/44)	*p* = 0.8
1	23.1% (*n* = 15/65)	16% (*n* = 7/44)	
2	15.4% (*n* = 10/65)	13.6% (*n* = 6/44)	
3	6.1% (*n* = 4/65)	11.4% (*n* = 5/44)	
Number of recurrences (GOSH: *N* = 71; USP: *N* = 52)*	1.4 ± 1.2	1.2 ± 1.5	*p* = 0.84
Timing for first recurrence (GOSH: *N* = 71; USP: *N* = 52)*	35 ± 59 months	13 ± 25.5 months	*p* = 0.013
Follow-up (years) (GOSH: *N* = 71; USP: *N* = 52)*	7.5 ± 4.8 years	10.1 ± 5.6 years	

*GOSH*, Great Ormond Street Hospital/*USP*, Universidade de São Paulo/*DDAVP*, desmopressin

^*^Denominators vary due to missing data; *n*/*N* shown per variable

### Recurrence

**Fig. 5:**
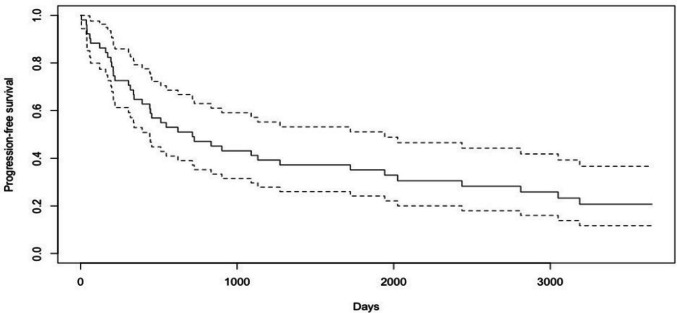
Progression-free survival of combined multi-centre craniopharyngioma cohort (*n* = 123). Continuous line: mean progression-free survival/interrupted line: confidence interval 95%

Using the RAPNO criteria for recurrence as a cystic increase with neurological symptoms. There were no significant differences in the number of recurrences at the two sites (GOSH: 1.4 ± 1.2; USP: 1.2 ± 1.5; *p* < 0.8). Patients at GOSH had earlier recurrences (25.5 ± 32 months) compared to patients treated at USP (37.4 ± 59.5 months) (*p* = 0.01) (Fig. 5).

In a subgroup analysis of patients who received adjuvant radiotherapy as a component of their initial treatment (within 90 days after surgery; GOSH only, *n* = 25), 20% experienced long-term recurrence, with a median time to recurrence of 43 months (range 20–87 months). When evaluating overall recurrence in the whole GOSH cohort following RT (as adjuvant: < 90 days after first intervention; and as a subsequent treatment: > 90 days after first intervention) 18 out of 80 patients (22.5%) required further surgical intervention due to tumor regrowth, with a median time to recurrence of 38 months (range 4.5–147.2 months).

In the multivariate analysis (*n* = 123), after adjusting for Puget’s classification grade and predominant tumor type (solid vs cystic), both subtotal/gross-total resection (OR 0.17; 95% CI 0.05–0.55; *p* < 0.01) and older age at diagnosis (OR 0.91; 95% CI 0.82–0.99; *p* = 0.04) were associated with longer progression-free survival. No mortality was documented during follow-up in either patient series.

## Discussion

Comparative analysis of two large series of ACP in children identified different paradigms of surgical management driven by distinct clinical-radiological presentations. In the USP cohort, patients showed a trend toward later clinical presentation with a higher frequency of visual deficits, growth deficiency, and larger tumour cyst size on volumetric analysis. By contrast, there were significantly higher rates of hypothalamic involvement (grade 2) in the tumours presenting to GOSH, which led to a different surgical strategy. More than 60% of patients at GOSH underwent cyst aspiration followed by radiotherapy as initial treatment, while at USP, 90% underwent initial surgical debulking. Rates of GTR were similar in both cohorts. Long-term outcomes were comparable, although with a lower incidence of hypothalamic syndrome at GOSH. Multivariate analysis demonstrated longer PFS for children who underwent GTR and those presenting at an older age. In this observational comparison, minimally invasive surgical strategies combined with radiation therapy were associated with more favourable hypothalamic outcomes at follow-up; however, these comparisons reflect real-world practice, with differences in tumour characteristics, treatment pathway from different centres, and follow-up duration; therefore, they might not permit causal inference regarding the superiority of one treatment management to another.

### Strategies of surgical management

As suggested by Van Schaik et al.[3], radiological grading systems are still a critical tool for decision-making in the management of ACP. Given the critical importance of hypothalamic injury to long-term outcomes, the Paris classification[15] and Muller’s grading system[6] for hypothalamic involvement represent the most frequently used radiological guides to inform surgical approaches. In this study, radiological grading led to different surgical approaches, with more tumour debulking in the Brazilian cohort guided by less hypothalamic involvement on imaging, and more cyst decompression followed by radiotherapy in the GOSH series due to significantly higher hypothalamic involvement pre-operatively.

Larger published series of childhood ACP have shown a significant correlation between body mass index and the radiological grading of hypothalamic invasion[1, 3]. Additionally, clinical evidence of hypothalamic dysfunction pre-operatively is highly predictive of worse hypothalamic outcomes[3]. This underscores the importance of detailed clinical assessment and experienced neuroradiology input in determining the optimal surgical strategy and for counselling patients and families with regard to surgical risk and functional outcomes.

In this study, we combined two ACP cohorts from high-volume paediatric neurosurgery units with similar age and gender distribution. There was no difference in overall survival and number of recurrences comparing both series. We highlight that a comparative analysis of PFS using the RAPNO criteria might have limitations due to cases of cyst regrowth that required repeated aspirations.

On multivariate analysis of the combined cohort (*n* = 123), tumour debulking (ST or GTR) and older age were predictors of longer PFS. While it is acknowledged that there is a benefit to offer tumour debulking aiming for GTR in selected cases[17–19], the optimal surgical approach should be decided through multidisciplinary discussion, with an emphasis on minimising hypothalamic morbidity[3]. The endoscopic endonasal approach has expanded surgical options for selected suprasellar tumours; however, its applicability is limited in lesions with marked lateral and high cystic extension or hypothalamic involvement[17]. Intracystic therapies also represent an important contemporary cyst-directed option in paediatric ACP management. These approaches were not analysed in detail in the present study, and a focused evaluation of cyst-directed treatments will be reported separately[20]. In the analysis of a subgroup of patients who underwent RT as either adjuvant or subsequent treatment, the median time to recurrence was longer compared to those who underwent surgical debulking alone. Less invasive surgical techniques, advanced MR analysis, and the clinical expertise of a team of experts likely provide the best outcomes for patients with ACP.

### Long-term clinical outcomes

Based on our previously described grading system[13], there were similar outcomes regarding pituitary and cognitive functions in the two cohorts. However, patients treated at GOSH had more favourable hypothalamic scores compared to USP. Whilst there were higher rates of hypothalamic involvement on baseline MR imaging, hypothalamic scores were more favourable in the GOSH cohort. This may reinforce differences in treatment approach, but also potential confounding by baseline tumour features, post-treatment endocrine resources, and follow-up of patients.

### Hypothalamic morbidity

We are not aware of any studies where a direct comparison between two cohorts of children with ACP, treated contemporaneously, evaluated using the same standardised outcomes. This study supports previous evidence of higher hypothalamic morbidity with more invasive approaches[1, 21]. When comparing the two cohorts, the main difference was observed in patients classified as grade 4—the most severe form of hypothalamic injury—which was more prevalent in the USP cohort. Despite the higher overall rates of hypothalamic involvement in the GOSH cohort, outcomes were more favourable in this group, which underwent less invasive surgical management. Although there has been some success with early, aggressive, and multidisciplinary management, involving endocrinological, dietary, rehabilitative, and psychological care [1, 3], hypothalamic injury remains a challenging condition for the physician and a life-altering condition for the patient and their families. It is important to highlight the importance of optimised post-operative clinical management paradigms in long-term outcomes related to BMI, as they may not be directly caused by variations in surgical approaches alone [3,. Furthermore, multicentre prospective studies are necessary to explore the potential for these measurement biases.

### Pituitary Dysfunction

While there were similar rates of panhypopituitarism between the two cohorts at the latest follow-up, more patients at GOSH received thyroid and growth hormone replacement. In multivariate analysis, there were no variables predicting endocrine outcomes. This difference might be explained by different follow-up duration between series and may potentially also be related to detection bias. Most patients at GOSH were still within the paediatric age range and were therefore receiving growth hormone replacement. Optimal replacement of growth hormones is critical to reach the expected stature in pediatric patients[4]. A recent study provides evidence for safety and pituitary function preservation after radiotherapy for ACP[21]. Studies with longer follow-up, including patients followed beyond the age of transition, are now necessary, including data on the final versus expected statures for patients with ACP in order to evaluate the impact of contemporary treatment strategies on endocrine outcome. Detection bias may have influenced the differences observed between series, potentially accounting for the higher pre-operative rate of GH deficiency at USP; in addition to that, the higher frequency of GH replacement at GOSH is likely explained by better availability and funding for hormone therapy for longer periods.

### Role of local invasiveness by the tumour

We found a high prevalence of DI in both cohorts. In this study, outcomes on DI and vision appear unrelated to surgical approaches but rather more related to direct involvement of the hypothalamus by the tumour. This might be the result of the inflammatory nature of ACP within the solid and cystic components, which have elucidated mechanisms of cytokine secretion triggering mechanisms of tumour invasion [6, 22–24]. This hypothesis has also been supported by the visual outcomes presented in this study. Favourable outcomes with long-term improvement in visual field deficits or acuity have not been noted, neither with surgical decompression nor with less invasive treatments for CP. Previous studies have demonstrated that visual outcomes did not seem to change with different management or even with more dedicated multidisciplinary care [3]. Some studies have demonstrated visual improvement with the transsphenoidal approach [17,; however, those outcomes were reported during the early post-operative period (immediate to 3 months). In our study, patients were assessed at the latest follow-up, taking into consideration that most patients presented with multiple tumour recurrences and underwent multiple interventions. Future studies with close follow-up and prospective documentation of visual function are necessary to investigate visual deterioration by tumour invasion or treatment-related morbidity.

### Strengths and limitations

There are a number of limitations to this study. Data were collected retrospectively and were dependent on the quality of clinical documentation. Importantly, this inter-cohort comparison includes substantial heterogeneity in treatment strategies, follow-up duration, and healthcare system context. Observed differences should be interpreted as associative and hypothesis-generating rather than causal and may reflect confounding by resource availability rather than intrinsic superiority of a single management decision-making.

Some subgroups were underpowered due to the retrospective nature of the series, which may have led to biased interpretations. Adjusted multivariable model could further explore whether management strategy independently influences hypothalamic outcome; however, this was not undertaken due to some constraints in the dataset. There was instead emphasised descriptive comparisons and clinically grounded stratification to avoid overinterpretation from an underpowered model. For all the children with incomplete documentation, a follow-up video or telephone consultation was performed to obtain more accurate details of the initial presentation. Radiological analysis and data collection were performed by the same neuroradiologist and neurosurgeon in both series to avoid measurement bias; however, limitations inherent to the radiological grading system itself should also be taken into consideration when interpreting differences between the GOSH and USP cohorts. Different institutions had different follow-up policies; those in Brazil were followed up until adulthood, while at GOSH they were transitioned to adult centres by the age of 18 years. This difference limited interpretations on some functional outcomes such as requirement for hormone replacement, as well as different criteria for diagnostic testing and hormone replacement. Follow-up was not long enough to allow documentation of late hormone deficits following radiotherapy, delayed hypothalamic dysfunction, long-term hypothalamic comorbidities (e.g. stroke, heart diseases), late effects of radiotherapy such as vasculopathy and new tumours or late ACP recurrence. It is important to highlight that clinical management of patients after surgery also significantly impacts hypothalamic outcomes. Indeed, patients in the UK have a wider range of treatment available for hypothalamic obesity (e.g., GLP-1 agonists, rehabilitation, psychological support—resource-related confounding) that could enable better outcomes. However, our series represents the longest follow-up of childhood CP with valuable insights on comparative analysis of cohorts from two paediatric units.

## Conclusions

Comparative analysis of two large paediatric ACP cohorts identified different paradigms of surgical management driven by different clinical-radiological presentations with broadly similar long-term outcomes. Better hypothalamic outcomes were associated with less invasive surgical treatment. These observational findings might not be causal and may be influenced by indications, referral patterns, follow-up duration, and healthcare system resources. GTR was related to better progression-free survival. More long-term longitudinal and comparative studies of ACP cohorts are needed to further define the long-term recurrence rate associated with different treatment paradigms, the potential risk of developing delayed post-treatment hypothalamic injury, and the potential long-term risks associated with proton therapy.

## Data Availability

No datasets were generated or analysed during the current study.
